# Nationwide analysis of antimicrobial resistance in pathogenic *Escherichia coli* strains isolated from diseased swine over 29 years in Japan

**DOI:** 10.3389/fmicb.2023.1107566

**Published:** 2023-03-17

**Authors:** Masahiro Kusumoto, Yukino Tamamura-Andoh, Yuna Hikoda-Kogiku, Asami Magome, Erina Okuhama, Keisuke Sato, Yoshino Mizuno, Nobuo Arai, Ayako Watanabe-Yanai, Taketoshi Iwata, Yoshitoshi Ogura, Yasuhiro Gotoh, Keiji Nakamura, Tetsuya Hayashi, Masato Akiba

**Affiliations:** ^1^National Institute of Animal Health, National Agriculture and Food Research Organization, Tsukuba, Japan; ^2^Graduate School of Veterinary Science, Osaka Metropolitan University, Osaka, Japan; ^3^Ehime Chuyo Livestock Hygiene Service Center, Ehime, Japan; ^4^Kagoshima Central Livestock Hygiene Service Center, Kagoshima, Japan; ^5^Miyazaki Livestock Hygiene Service Center, Miyazaki, Japan; ^6^Niigata Chuo Livestock Hygiene Service Center, Niigata, Japan; ^7^Kumamoto Chuo Livestock Hygiene Service Center, Kumamoto, Japan; ^8^Department of Infectious Medicine, Kurume University School of Medicine, Fukuoka, Japan; ^9^Department of Bacteriology, Faculty of Medical Sciences, Kyushu University, Fukuoka, Japan; ^10^School of Veterinary Medicine, Rakuno Gakuen University, Ebetsu, Japan

**Keywords:** apramycin, bicozamycin, multidrug-resistant, pathogenic *Escherichia coli*, swine

## Abstract

Pathogenic *Escherichia coli* strains are important causes of several swine diseases that result in significant economic losses worldwide. In Japan, the use of antimicrobials in swine is much higher than that in other farm animals every year. Antimicrobial resistance in pathogenic *E. coli* strains also heavily impacts the swine industry due to the limited treatment options and an increase in the potential risk of the One Health crisis. In 2016, we investigated 684 Japanese isolates of swine pathogenic *E. coli* belonging to four major serogroups and reported the emergence and increase in the highly multidrug-resistant serogroups O116 and OSB9 and the appearance of colistin-resistant strains. In the present study, by expanding our previous analysis, we determined the serotypes and antimicrobial resistance of 1,708 *E. coli* strains isolated from diseased swine between 1991 and 2019 in Japan and found recent increases in the prevalences of multidrug-resistant strains and minor serogroup strains. Among the antimicrobials examined in this study that have been approved for animal use, a third-generation cephalosporin was found to be effective against the most isolates (resistance rate: 1.2%) but not against highly multidrug-resistant strains. We also analyzed the susceptibilities of the 1,708 isolates to apramycin and bicozamycin, both which are available for treating swine in Japan, and found that the rates of resistance to apramycin and bicozamycin were low (6.7% and 5.8%, respectively), and both antimicrobials are more effective (resistance rates: 2.7% and 5.4%, respectively) than third-generation cephalosporins (resistance rate: 16.2%) against highly multidrug-resistant strains.

## 1. Introduction

Pathogenic *Escherichia coli* strains are important causes of several diseases in swine worldwide, including neonatal diarrhea, postweaning diarrhea, edema disease, and septicemia. In particular, diarrhea and edema disease result in significant economic losses due to high morbidity and mortality, decreased weight gain, and the cost of treatment (Fairbrother and Gyles, 2012; Abubakar et al., 2017). Antimicrobial resistance (AMR) of pathogenic *E. coli* also heavily impacts the swine industry because of the limited treatment options and an increased public health crisis due to the potential transfer of AMR genes into food chains (Fairbrother and Gyles, 2012). The emergence of multidrug-resistant (MDR) bacteria, which are defined as bacteria that are resistant to at least one antimicrobial agent in each of three or more drug classes (Magiorakos et al., 2012), are widely recognized as one of the most important current threats to public health (van Duin and Paterson, 2020). In Japan, the use of antimicrobial drugs in farm animals, which has been calculated from the sales volume of veterinary medical products, is highest in swine, followed by poultry and cattle (Hosoi et al., 2013). We previously determined the susceptibilities of the *E. coli* strains O139, O149, O116, and OSB9 (684 in total), which represent the four major serogroups of *E. coli* strains isolated between 1991 and 2014 from diseased swine in Japan, to 21 antimicrobials selected from various drug classes and found that most of the 684 strains (91%) were resistant to one or more antimicrobial (Kusumoto et al., 2016a). The O116 and OSB9 strains showed resistance to a wider range of antimicrobials than the O139 and O149 strains, and surprisingly, all and 68% of the O116 and OSB9 strains, respectively, were resistant to fluoroquinolones (FQs) (Kusumoto et al., 2016a). We also investigated the susceptibility of these 684 strains to colistin and demonstrated that 309 (45%) were colistin resistant (Kusumoto et al., 2016b). The rates of colistin resistance among the O139, O149, O116, and OSB9 strains did not differ significantly. Because the isolation rate of colistin-resistant *E. coli* from healthy animals has been low (1%) in Japan (Suzuki et al., 2016), the recent emergence of colistin-resistant swine pathogenic *E. coli* was considered to be associated with the use of colistin to treat swine diseases. However, the AMR of swine pathogenic *E. coli* strains other than the four major serogroups remains unclear.

Colistin has recently become an important antimicrobial in human medicine for the treatment of infectious diseases caused by MDR Gram-negative bacteria such as *Pseudomonas aeruginosa*, *Acinetobacter baumannii*, and carbapenem-resistant *Enterobacteriaceae* (Ruppe et al., 2015). In contrast, colistin has been used in veterinary medicine for several decades, not only for the prevention and treatment of infectious diseases but also for growth promotion (Kempf et al., 2016). With the emergence of colistin resistance gene (*mcr-1*)-positive bacteria, its use in animals has been re-evaluated in many countries (Shen et al., 2020). Notably, the Food Safety Commission of Japan conducted a risk assessment in 2017 focusing on antimicrobial-resistant *E. coli* arising from the use of colistin (Food Safety Commission Japan, 2017). Based on this assessment, the Ministry of Agriculture, Forestry and Fisheries of Japan prohibited the use of feed additives, including colistin, and transferred colistin from a first-choice to second-choice drug for the treatment of diseased swine in 2018 (Makita et al., 2020). FQs are also regarded as second-choice drugs for swine in Japan because of their importance in human medicine, and FQ resistance has essentially remained at a low level in commensal *E. coli* isolated from healthy swine in Japan (Harada and Asai, 2010). As such, the emergence of *E. coli* strains O116 and OSB9 that are resistant to multiple drugs, including FQs, and colistin-resistant pathogenic *E. coli* strains are significant risks to animal production. Extensive assessment of the risk of antimicrobial use to select resistant *E. coli* strains and the development of novel antimicrobial agents effective against resistant strains must control the emergence and spread of AMR strains in both humans and animals under the One Health concept.

In the present study, we collected more extensive data on the AMR of swine pathogenic *E. coli* than that gathered in previous studies in terms of the number of isolates (1,708 strains) and antimicrobial agents (24 antimicrobials, which included the previously analyzed 22 antimicrobials plus two types of combination drugs composed of β-lactam and β-lactamase inhibitors), to obtain a wider view on the spread of AMR among these strains in Japan. The susceptibility of the 1,708 strains to apramycin and bicozamycin (previously known as bicyclomycin) was also analyzed to evaluate the effectiveness of these antimicrobials against swine pathogenic *E. coli*.

## 2. Materials and methods

### 2.1. Bacterial strains and culture conditions

We investigated 1,708 *E. coli* strains that were isolated in Japan between 1991 and 2019 from swine that died of neonatal diarrhea, postweaning diarrhea, or edema disease (Table 1; see also Supplementary Table 1 for more detailed information), and 967 of these strains were described in our previous studies (Kusumoto et al., 2016a,b). These strains represent all of the swine disease-associated *E. coli* strains isolated from the 31 Japanese prefectural Livestock Hygiene Service Centers during this period. Samples collected by the prefectural centers were sent to the National Institute of Animal Health for diagnostic purposes. The bacterial strains were identified as *E. coli* by biochemical tests using the API20E system and the apiweb database (Sysmex-bioMerieux, Tokyo, Japan) according to the manufacturer’s instructions. The O serogroups of the strains were determined with agglutination tests using antisera that were obtained from the Statens Serum Institute (Copenhagen, Denmark) or Denka Seiken Co., Ltd. (Tokyo, Japan) according to the manufacturer’s instructions. All strains were grown in Mueller-Hinton broth (Becton, Dickinson and Company, Sparks, MD) for 18 h at 37°C.

**TABLE 1:** Serogroups of *E. coli* isolates from swine in Japan between 1991 and 2019.

	No. of isolates			
	**Reported case**			
**O serogroup^a^**	**Diarrhea**	**Edema disease**	**Others/Unknown**	**Total (%)**
O139	40	389	12	441 (25.8)
O149	333	2	13	348 (20.4)
O116	199	32	2	233 (13.6)
OSB9	106	23	4	133 (7.8)
O8	31	9	3	43 (2.5)
O141	8	35	0	43 (2.5)
O2	9	14	11	34 (2.0)
O147	31	0	3	34 (2.0)
O9	19	2	5	26 (1.5)
O86	11	6	1	18 (1.1)
O45	13	0	4	17 (1.0)
O121	4	7	3	14 (0.8)
O142	1	4	9	14 (0.8)
O180	11	2	0	13 (0.8)
O35	11	1	0	12 (0.7)
O103	4	0	7	11 (0.6)
O157	9	2	0	11 (0.6)
O98	5	3	0	8 (0.5)
O138	8	0	0	8 (0.5)
O115	3	0	3	6 (0.4)
O15	5	0	0	5 (0.3)
O20	3	0	2	5 (0.3)
O51	1	2	2	5 (0.3)
O1	0	4	0	4 (0.2)
O7	4	0	0	4 (0.2)
O25	3	0	1	4 (0.2)
O36	3	0	1	4 (0.2)
O108	2	1	1	4 (0.2)
O159	4	0	0	4 (0.2)
O4	0	0	3	3 (0.2)
O26	2	0	1	3 (0.2)
O55	1	2	0	3 (0.2)
O64	3	0	0	3 (0.2)
O84	2	1	0	3 (0.2)
O132	2	0	1	3 (0.2)
O6	0	2	0	2 (0.1)
O16	2	0	0	2 (0.1)
O23	2	0	0	2 (0.1)
O28	1	0	1	2 (0.1)
O68	2	0	0	2 (0.1)
O76	1	0	1	2 (0.1)
O77	1	1	0	2 (0.1)
O78	1	0	1	2 (0.1)
O83	0	1	1	2 (0.1)
O112	2	0	0	2 (0.1)
O160	2	0	0	2 (0.1)
O162	1	0	1	2 (0.1)
O175	2	0	0	2 (0.1)
O10	1	0	0	1 (0.1)
O29	1	0	0	1 (0.1)
O43	1	0	0	1 (0.1)
O66	0	0	1	1 (0.1)
O75	1	0	0	1 (0.1)
O88	1	0	0	1 (0.1)
O89	0	0	1	1 (0.1)
O91	0	0	1	1 (0.1)
O114	1	0	0	1 (0.1)
O118	1	0	0	1 (0.1)
O127	0	0	1	1 (0.1)
O128	1	0	0	1 (0.1)
O154	0	0	1	1 (0.1)
O161	1	0	0	1 (0.1)
O163	1	0	0	1 (0.1)
O166	0	0	1	1 (0.1)
O174	0	0	1	1 (0.1)
O177	0	0	1	1 (0.1)
O186	1	0	0	1 (0.1)
OSB17	1	0	0	1 (0.1)
OSD10	0	0	1	1 (0.1)
OUT	88	22	27	137 (8.0)
Total	1,008	567	133	1,708 (100)

^a^SB9, *S. boydii* type 9; SB17, *S. boydii* type 17; SD10, *S. dysenteriae* type 10; UT, untypable.

### 2.2. Antimicrobial susceptibility testing

The 0.5 McFarland test strain suspensions were prepared using the Prompt System (Becton, Dickenson and Company). The following 23 antimicrobials were tested by the Kirby-Bauer disc diffusion test using Sensi-Disc Susceptibility Discs (Becton, Dickinson and Company) according to the recommendation of the Clinical and Laboratory Standards Institute (CLSI) (ClSI, 2016): ampicillin (10 μg), piperacillin (100 μg), cefazolin (30 μg), cefuroxime (30 μg), cefotaxime (30 μg), cefepime (30 μg), cefoxitin (30 μg), moxalactam (30 μg), aztreonam (30 μg), imipenem (10 μg), meropenem (10 μg), gentamicin (10 μg), kanamycin (30 μg), streptomycin (10 μg), tetracycline (30 μg), chloramphenicol (30 μg), nalidixic acid (30 μg), ciprofloxacin (5 μg), levofloxacin (5 μg), gatifloxacin (5 μg), trimethoprim-sulfamethoxazole (1.25/23.75 μg), ampicillin-sulbactam (10/10 μg), and piperacillin-tazobactam (100/10 μg). We included multiple antimicrobials from each drug class, as bacterial susceptibilities to antimicrobial agents often differ, even among agents belonging to the same class. The MICs of the other three antimicrobials, colistin (Merck KGaA, Darmstadt, Germany), apramycin (Merck), and bicozamycin (MSD Animal Health K.K., Osaka, Japan), were determined by the agar dilution method in accordance with the guidelines of the CLSI (2015). The isolates with a colistin MIC of ≥ 4 μg/ml were considered colistin resistant based on the European Committee on Antimicrobial Susceptibility Testing (EUCAST) criteria (EUCAST, 2021). When the MICs for apramycin and bicozamycin showed bimodal distribution, the breakpoints were set as the midpoint between the two peaks of each MIC distribution by reference to the previous report (Esaki et al., 2004). The *E. coli* reference strain ATCC 29522 was used as a quality control in the MIC determinations.

The AMR of 684 strains for 22 antimicrobials (ampicillin, piperacillin, cefazolin, cefuroxime, cefotaxime, cefepime, cefoxitin, moxalactam, aztreonam, imipenem, meropenem, gentamicin, kanamycin, streptomycin, tetracycline, chloramphenicol, nalidixic acid, ciprofloxacin, levofloxacin, gatifloxacin, trimethoprim-sulfamethoxazole, and colistin) was determined in our previous studies (Kusumoto et al., 2016a,b), and these data were integrated with the data determined in the present study.

### 2.3. Statistical analysis

The statistical significance of the observed differences in the percentages of the strains that were resistant to each antimicrobial agent was analyzed by Fisher’s exact test with Bonferroni correction and was calculated by EZR software (Kanda, 2013).

## 3. Results

### 3.1. Prevalence of O serogroups in swine pathogenic *E. coli* isolates

We analyzed 1,708 *E. coli* strains isolated from diseased swine in Japan between 1991 and 2019 (Table 1; see also Supplementary Table 1 for more details). Among the 1,708 strains, the O serogroups of 967 strains were determined in our previous study (Kusumoto et al., 2016a), and those of the remaining 741 strains were determined in this study. As shown in Table 1, 1,571 strains (92.0%) were classified into 69 O serogroups, while 137 (8.0%) were untypeable (OUT). O139 (25.8%), O149 (20.4%), O116 (13.6%), and OSB9 (7.8%) were predominant among the 69 serogroups, together representing 67.6% of the 1,708 strains examined.

**FIGURE 1:**
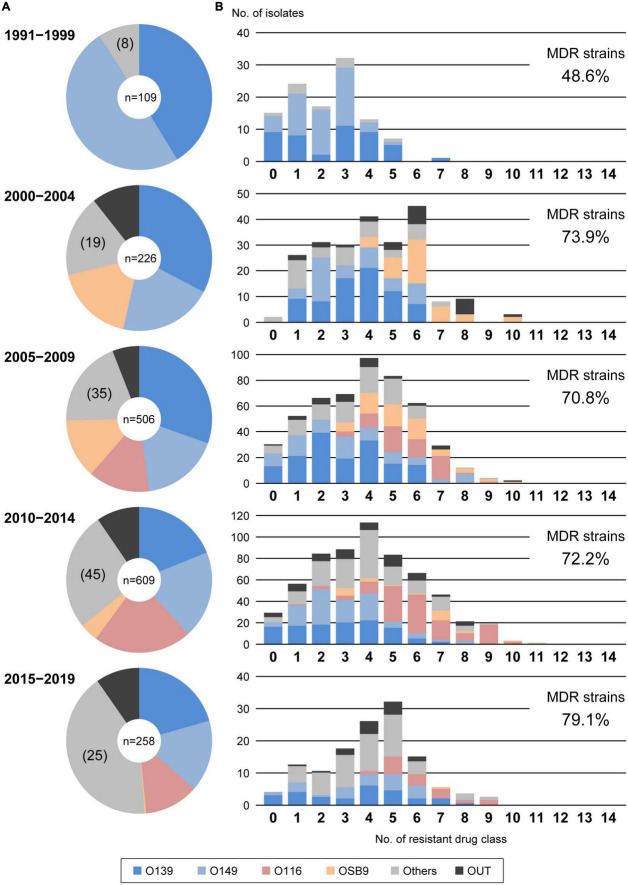
Temporal changes in O serogroups **(A)** and multidrug resistance levels among each O serogroup **(B)** in *E. coli* isolated from diseased swine in Japan. The O serogroup prevalences and multidrug resistance levels between 1991 and 1999, 2000 and 2004, 2005 and 2009, 2010 and 2014, and 2015 and 2019 were compared. O serogroups are indicated with the following colors: blue, O139; light blue, O149; pink, O116; orange, OSB9; dark gray, untypeable (OUT); and gray, O serogroups other than O139, O149, O116, OSB9, and OUT.

Figure 1A shows the temporal changes in the prevalence of O serogroups between 1991 and 2019. O139 and O149, the two most predominant O serogroups, first appeared in 1991 and 1993, respectively, and their proportions were very high in the period from 1991 to 1999 (41.3% and 49.5%, respectively), but their proportions have gradually decreased over time (20.5% and 15.9%, respectively, in the period from 2015 to 2019). The proportions of O116 and OSB9, which first appeared in 2005 and 2000, respectively, were initially high but have decreased in recent years. In contrast, the proportions of other minor O serogroups have gradually increased in recent years, accounting for 41.5% of the strains isolated in the period from 2015 to 2019. In particular, O141, O2, O9, O86, O45, O142, O103, and O115 exhibited notable increases in prevalence (Supplementary Figure 1).

### 3.2. Antimicrobial susceptibility and multidrug resistance

We determined the susceptibility of the 1,708 strains to 24 antimicrobials and analyzed the proportion of strains resistant to each antimicrobial in the entire strain set (Supplementary Table 1). While no strains showed resistance to moxalactam, imipenem, meropenem, or piperacillin-tazobactam, 1,643 strains (96.2%) were resistant to one or more of the 24 antimicrobials. The rates of resistance to cefuroxime, cefotaxime, cefepime, and aztreonam in the entire strain set were extremely low (3.5%, 1.2%, 0.2%, and 1.0%, respectively). In contrast, more than half of the strains showed resistance to six important veterinary antimicrobials: ampicillin (60.5%), streptomycin (58.6%), tetracycline (77.2%), chloramphenicol (54.7%), trimethoprim-sulfamethoxazole (52.6%), and colistin (52.8%).

**FIGURE 2:**
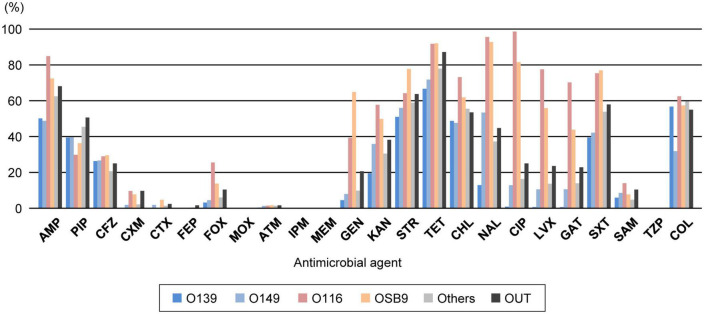
Comparison of the prevalence of O serogroups and rates of resistance to each antimicrobial. AMP, ampicillin; PIP, piperacillin; CFZ, cefazolin; CXM, cefuroxime; CTX, cefotaxime; FEP, cefepime; FOX, cefoxitin; MOX, moxalactam; ATM, aztreonam; IPM, imipenem; MEM, meropenem; GEN, gentamicin; KAN, kanamycin; STR, streptomycin; TET, tetracycline; CHL, chloramphenicol; NAL, nalidixic acid; CIP, ciprofloxacin; LVX, levofloxacin; GAT, gatifloxacin; SXT, trimethoprim-sulfamethoxazole; SAM, ampicillin-sulbactam; TZP, piperacillin-tazobactam; COL, colistin. O serogroups are indicated with the same colors as those in Figure 1.

Analysis of the interserogroup differences in the proportions of strains resistant to each antimicrobial (Figure 2; see also Supplementary Figure 2 for the statistical significance of the differences between O139, O149, O116, and OSB9) revealed that the resistance profiles of O139 and O149 are similar in general, but the O149 strains showed significantly higher resistance rates to seven antimicrobials (cefuroxime, cefotaxime, kanamycin, nalidixic acid, ciprofloxacin, levofloxacin, and gatifloxacin; *P* < 0.05) and lower rates to colistin (*P* < 0.001). The profiles of O116 and OSB9 were also similar, but the O116 strains showed higher resistance rates to five antimicrobials (ampicillin, cefoxitin, ciprofloxacin, levofloxacin, and gatifloxacin; *P* < 0.05) and lower resistance rates to three antimicrobials (cefotaxime, gentamicin, and streptomycin; *P* < 0.05). Compared to both O139 and O149, O116 and OSB9 showed higher resistance rates to 11 antimicrobials (*P* < 0.05): ampicillin, cefuroxime, cefoxitin, gentamicin, kanamycin, tetracycline, nalidixic acid, ciprofloxacin, levofloxacin, gatifloxacin, and trimethoprim-sulfamethoxazole (Figure 1 and Supplementary Figure 1). Notable differences were observed for FQ resistance between O139/O149 and O116/OSB9 (P < 0.001), while only 0.5-0.7% and 10.3-12.6% of the O139 and O149 strains were resistant to the three FQs examined, respectively, and 70.0-98.3% and 43.6-81.2% of the O116 and OSB9 strains were resistant to these FQs, respectively.

**FIGURE 3:**
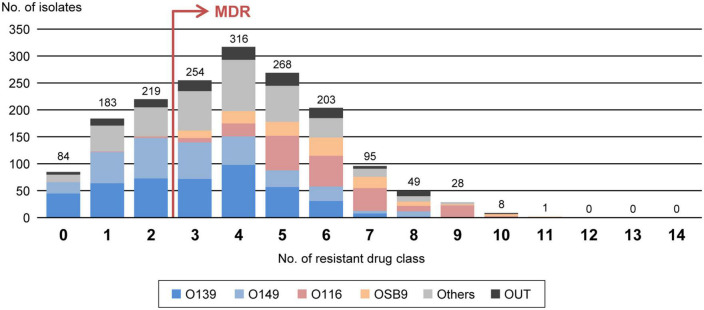
Comparisons of multidrug resistance levels among each O serogroup. The *x*-axis shows the number of drug classes to which the isolates in the serogroups are resistant. MDR isolates are defined as isolates that are resistant to three or more drug classes, as indicated by the red arrow. The numbers of isolates presenting resistance to each drug class are indicated above the bars. O serogroups are indicated with the same colors as those in Figure 1.

According to the proposal by Magiorakos et al. (2012), we also evaluated the multidrug resistance of the 1,708 strains by counting the number of strains resistant to 14 antimicrobial drug classes (Supplementary Table 2) and identified 1,222 MDR *E. coli* strains (71.5%) resistant to three or more drug classes (Figure 3). The numbers of drugs to which the O116 and OSB9 strains showed resistance were significantly higher (median of 6 classes for each) than those for the O139 and O149 strains (median of 3 classes for each) (*P* < 0.001). Surprisingly, the prevalence of O116 MDR strains was 99.6%, and all OSB9 strains were MDR. Furthermore, 24 O116 (64.9%) and seven OSB9 (18.9%) strains were resistant to more than nine of the 14 drug classes, whereas the O139 and O149 strains did not show extremely high levels of multidrug resistance (Supplementary Table 3). The temporal changes in the level of multidrug resistance between 1991 and 2019 shown in Figure 1B indicate that there were fewer MDR strains in the period from 1991 to 1999 (48.6%) than the number of MDR strains isolated later than 2000 (70.8-79.1%). This increase in the prevalence of MDR strains is mainly attributable to the increase in MDR O116/OSB9 strains, but it is also notable that several minor O serogroups showed a high prevalence of MDR strains: 86.0% (O141), 97.1% (O147), 88.9% (O86), 84.6% (O180), 91.7% (O35), 81.8% (O103), 81.8% (O157), and 85.7% (O98) (Supplementary Figure 1). In addition, notable differences were observed for the temporal change in resistance rate between antimicrobials (Supplementary Figure 3). The prevalence of gentamicin-, nalidixic acid-, FQs (ciprofloxacin, levofloxacin, and gatifloxacin)-, trimethoprim- sulfamethoxazole-, and colistin-resistant strains has also significantly increased after 2000.

### 3.3. Apramycin and bicozamycin effectiveness against MDR strains

**FIGURE 4:**
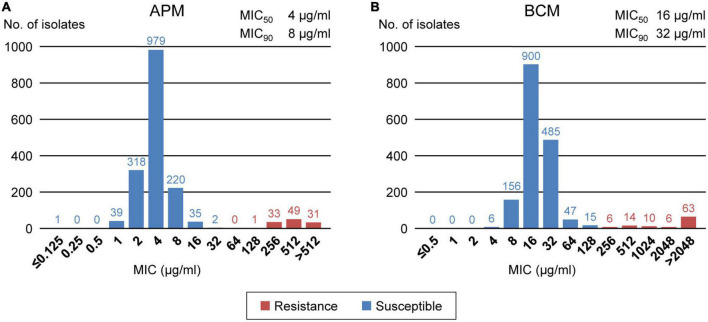
Distributions of the MICs of apramycin **(A)** and bicozamycin **(B)** for the 1,708 *E. coli* isolates from diseased swine in Japan between 1991 and 2019. The numbers of isolates presenting with each MIC are indicated above the bars. The susceptible and resistant isolates are shown in blue and red, respectively. The MIC50 and MIC90 values are shown at the upper right of each panel.

We analyzed the apramycin and bicozamycin resistance of the 1,708 strains by determining their MICs in the range of 0.125-512 μg/ml for apramycin and 0.5-2,048 μg/ml for bicozamycin (Figure 4). The MICs against apramycin showed bimodal distribution with two peaks at 4 and 512 μg/ml (Figure 4A). When the breakpoint was set to 64 μg/ml, the midpoint between the two peaks, 114 (6.7%) of the 1,708 strains were classified as apramycin resistant (Table 2). The MIC_50_ and MIC_90_ values were 4 and 8 μg/ml, respectively. The MICs against bicozamycin also showed bimodal distribution with two peaks at 16 and > 2,048 μg/ml (Figure 4B; note that it was difficult to test concentrations higher than 2,048 μg/ml due to the solubility of bicozamycin). Therefore, a breakpoint for bicozamycin was set to 256 μg/ml, the midpoint between the two peaks. With this breakpoint, 99 (5.8%) of the 1,708 strains were classified as bicozamycin resistant (Table 2). The MIC_50_ and MIC_90_ values were 16 and 32 μg/ml, respectively. Apramycin-resistant strains generally showed higher levels of multidrug resistance than bicozamycin-resistant strains, i.e., the median values of the drug classes to which the apramycin strains were resistant were significantly higher than those in bicozamycin-resistant strains (6 classes vs. 4 classes; *P* < 0.001). More importantly, there were only six strains (0.4%) resistant to both apramycin and bicozamycin in the strain set, meaning that the other 1,702 strains (99.6%) were susceptible to apramycin, bicozamycin, or both (Table 2).

**TABLE 2:** Distributions of apramycin- and/or bicozamycin-resistant isolates in each number of resistant drug class.

		No. of isolates															
**Resistant (R) or susceptible (S) to^a^**			**No. of resistant drug class**														
APM	BCM	Total (%)	0	1	2	3	4	5	6	7	8	9	10	11	12	13	14
R	R	6 (0.4)	0	0	0	3	0	1	0	2	0	0	0	0	0	0	0
R	S	108 (6.3)	0	2	2	4	11	24	45	12	7	0	1	0	0	0	0
S	R	93 (5.4)	6	8	21	10	19	8	10	6	3	0	2	0	0	0	0
S	S	1501 (87.9)	78	173	196	237	286	235	148	75	39	28	5	1	0	0	0

^a^APM, apramycin; BCM, bicozamycin.

## 4. Discussion

Through nationwide analysis of swine pathogenic *E. coli* strains isolated over 29 years (1991-2019) in Japan, we showed that there were temporal changes in the prevalence of four major serogroups and other serogroups and differences in the AMR profiles between serogroups (Figure 2). O139 and O149 have gradually decreased, and two later appearing serogroups (O116 and OSB9) once became dominant, but their prevalence has also decreased recently. However, these four major serogroups are still important swine pathogens because the O139, O149, and O116 strains are still predominant (altogether representing nearly half of the strains isolated between 2015 and 2019; Figure 1), and O116 and OSB9 include strains with extremely high levels of multidrug resistance (Figure 3). Contrary to the recent decreases in these four serogroups, the prevalence of other minor serogroups has recently increased (representing 41.5% of the strains isolated between 2015 and 2019; Figure 1), indicating that more diverse *E. coli* strains have recently caused diseases in swine. Notably, many of these strains, e.g., O141, O147, O86, O180, O35, O103, O157, and O98, include a high prevalence of MDR strains (Supplementary Figure 1). In addition, strains showing the same high multidrug resistance levels as the O116 and OSB9 strains were found among the O86, O159, and OUT strains (Figure 3 and Supplementary Table 3). Therefore, continuous monitoring of the prevalence and AMR of not only the major serogroups but also minor serogroups will be needed. The choice and use of proper antimicrobials is also important to achieve the maximum therapeutic benefit while minimizing the selection of resistant bacteria.

In Japan, the following drug classes are not approved for animal use (Ministry of Agriculture, Forestry and Fisheries, 2019): fourth-generation cephalosporins, cephamycins, oxacephems, monobactams, carbapenems, and combinations of β-lactams and β-lactamase inhibitors. These classes include cefepime, cefoxitin, moxalactam, aztreonam, imipenem, meropenem, ampicillin-sulbactam, and piperacillin-tazobactam, which were analyzed in the present study. Therefore, cefuroxime and cefotaxime (second- and third-generation cephalosporins, respectively) are the only effective and approved antimicrobials among the 24 agents analyzed (Figure 2). Notably, the rate of resistance to cefotaxime among the 1,708 strains was extremely low (1.2%). However, in our recent study, we observed a clear association between the therapeutic use of third-generation cephalosporins by swine farms and the selection of swine pathogenic *E. coli* that show resistance to this and other classes of β-lactams (Misumi et al., 2021). Furthermore, some of the selected *E. coli* strains produce extended-spectrum β-lactamase (ESBL) encoded by genes such as *bla*_*CTX–M–14*_ and *bla*_*CTX–M–15*_, which have also been found in *E. coli* isolated from humans (23). ESBL-producing *E. coli* strains have increasingly been isolated from humans, animals, food, and the environment worldwide and have become a serious concern due to resistance to clinically important third- and fourth-generation cephalosporins (Dorado-Garcia et al., 2018; Bush and Bradford, 2020). Therefore, the use of third-generation cephalosporins is effective against swine pathogenic *E. coli* but poses a considerable risk to select highly MDR pathogenic *E. coli* strains.

The effective and approved antimicrobial candidates other than third-generation cephalosporins found in this study are apramycin and bicozamycin. Currently, apramycin and bicozamycin can be used in veterinary medicine in Japan, including to treat diseased swine (Ministry of Agriculture, Forestry and Fisheries, 2019). Apramycin is an aminocyclitol aminoglycoside with a unique chemical structure that differs from that of clinically relevant aminoglycosides (Juhas et al., 2019). Bicozamycin is a unique agent containing a diketopiperazine ring that is not found in any other antimicrobial (Bentley et al., 1993). Therefore, both compounds have a broad spectrum of action, including against MDR Gram-negative bacteria (Kohn and Widger, 2005; Wachino and Arakawa, 2012; Smith and Kirby, 2016; Kang et al., 2017). However, the criteria for determining resistance to them have not yet been defined, and their effectiveness against swine pathogenic *E. coli* has also yet to be investigated. We therefore investigated the prevalence of swine pathogenic *E. coli* resistance to these agents. We defined a breakpoint of apramycin at 64 μg/ml (Figure 4A) in the present study, and this breakpoint is reliable because the same concentration was reported by the National Veterinary Assay Laboratory (National Veterinary Assay Laboratory, 2009), Smith and Kirby (2016), and Yang et al. (2020). The National Veterinary Assay Laboratory also determined the MICs of *E. coli* strains against bicozamycin to be in the range of 0.125–512 μg/ml and set the breakpoint at 128 μg/ml (National Veterinary Assay Laboratory, 2009). We extended the range of MIC measurements to 2,048 μg/ml and found that resistance produced a broad distribution with a peak at > 2,048 μg/ml (Figure 4B). As it is difficult to analyze concentrations higher than that due to the solubility of bicozamycin, the breakpoint defined in the present study (256 μg/ml) is reasonable considering the measurable range of its MIC.

The prevalence of apramycin- and bicozamycin-resistant strains was low (6.7% and 5.8% among the total 1,708 strains, respectively). More importantly, both antimicrobials are effective against highly MDR pathogenic *E. coli* strains: the rates of resistance to apramycin and bicozamycin among the top 37 MDR isolates (Supplementary Table 3) were much lower (2.7% and 5.4%, respectively) than that the resistance to extended-spectrum cephalosporins (16.2%). In Japan, the sales volumes of apramycin and bicozamycin in 2019 were 2,228.8 kg and 0.2 kg, respectively (Ministry of Agriculture, Forestry and Fisheries, 2019). In contrast, the sales volumes of ampicillin, streptomycin, and oxytetracycline (a commonly used tetracycline for farm animals in Japan), to which many of the *E. coli* strains showed resistance (Figure 2), were 35.9 t, 27.2 t, and 169.8 t, respectively (Ministry of Agriculture, Forestry and Fisheries, 2019). Thus, apramycin and bicozamycin are currently good choices to treat *E. coli* infection in swine, particularly highly MDR strains. If the amounts of antimicrobials used in farm animals are estimated from these sales volumes, the effectiveness of apramycin and bicozamycin is due not only to their unique chemical structures but also their low usage on farms. In *E. coli*, the aminoglycoside 3-N-acetyltransferase type IV gene (*aac(3)-IV*) has been identified as an apramycin resistance gene (Hunter et al., 1993) and is carried by plasmids in most apramycin-resistant strains isolated from swine (Mathew et al., 2003, 2005) and humans (Zhang et al., 2009). Therefore, careful monitoring of the prevalence of *aac(3)-IV* in swine pathogenic *E. coli* strains is needed. In contrast, there are few reports of bicozamycin resistance genes. However, it should be noted that *bcr1* was found in an integrative and conjugative element in *P. aeruginosa* (Fonseca et al., 2015). The World Organization for Animal Health (OIE) and the World Health Organization (WHO) have included bicozamycin in the lists “veterinary important antimicrobial agents” and “antimicrobial classes currently not used in humans”, respectively (OIE, 2019; WHO, 2019), and no bicozamycin-based animal drug or feed additive is currently used in the USA (U.S. Food and Drug Administration, 2003) or EU (European Medicines Agency, 2019).

In conclusion, our nationwide analysis of swine pathogenic *E. coli* strains isolated over 29 years in Japan revealed a recent increase in the prevalence of MDR and minor serogroup strains. We also showed that among the approved antimicrobials in Japan, cefuroxime and cefotaxime are effective, but apramycin and bicozamycin are more effective against pathogenic *E. coli* strains, including those showing a high level of multidrug resistance. However, as heavy use of antimicrobials is an important risk factor for AMR (Paterson and Bonomo, 2005), apramycin and bicozamycin (and third-generation cephalosporins such as cefotaxime) should be used prudently to achieve the maximum therapeutic benefit while minimizing the selection of resistant bacteria. Careful monitoring of the use of these antimicrobials and their resistance in swine pathogenic *E. coli* are also needed.

## Data availability statement

The original contributions presented in this study are included in the article/Supplementary material, further inquiries can be directed to the corresponding author.

## Author contributions

MK conceived and designed the study and wrote the manuscript. YT-A, YH-K, AM, EO, KS, YM, NA, AW-Y, and TI performed the experiments. YO, YG, KN, TH, and MA supervised and made an intellectual contribution to the work. All authors were responsible for acquisition and analysis of data, commented on the draft, and approved the final version of the manuscript.
