# Supplementary material for: Nationwide analysis of antimicrobial resistance in pathogenic Escherichia coli strains isolated from diseased swine over 29 years in Japan
Source: Front Microbiol. 2023 Mar 17;14:1107566. doi: 10.3389/fmicb.2023.1107566 (PMC10065406; doi:10.3389/fmicb.2023.1107566)
Supplement: Supplementary file 3 [file Data_Sheet_1.docx]

Supplementary Material


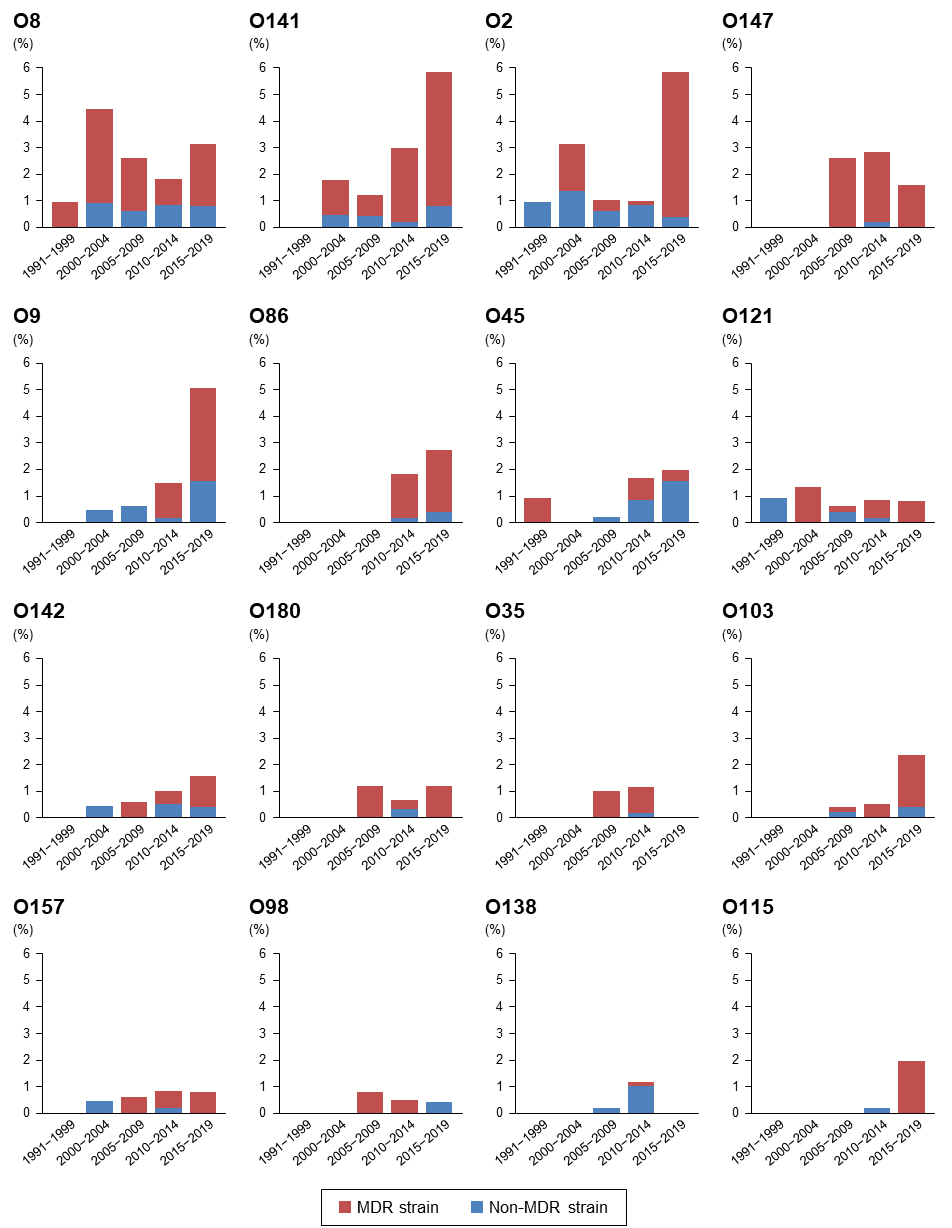


**Supplementary Figure 1.** Temporal changes in the prevalence and multidrug resistance of minor serogroups. The top 16 minor serogroups (those other than O139, O149, O116, OSB9, and OUT) that are shown in Table 1 were selected, and their prevalences between 1991 and 1999, 2000 and 2004, 2005 and 2009, 2010 and 2014, and 2015 and 2019 were compared. The multidrug-resistant and non-multidrug-resistant strains are shown in red and blue, respectively.


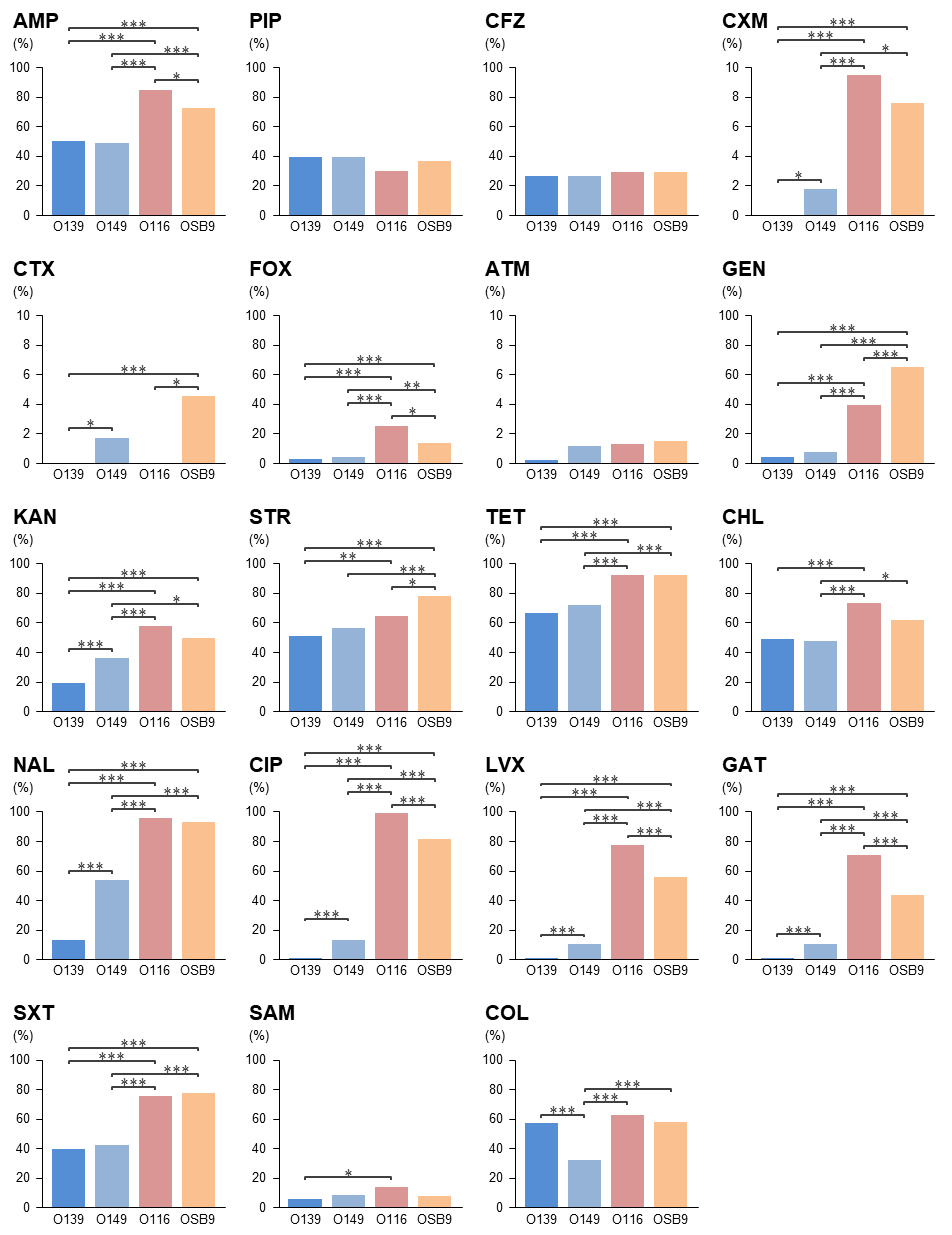


**Supplementary Figure 2.** Comparison of the rates of resistance to each antimicrobial among the four major serogroups of *E. coli* isolates from diseased swine in Japan. Asterisks indicate statistically significant differences between each combination of serogroups (Fisher's exact test with Bonferroni correction; *, P < 0.05; **, P < 0.01; ***, P < 0.001). O serogroups are indicated with the same colors as those in Figure 1 in the main text. Abbreviations for the antimicrobials are listed in the legend of Figure 2 in the main text.


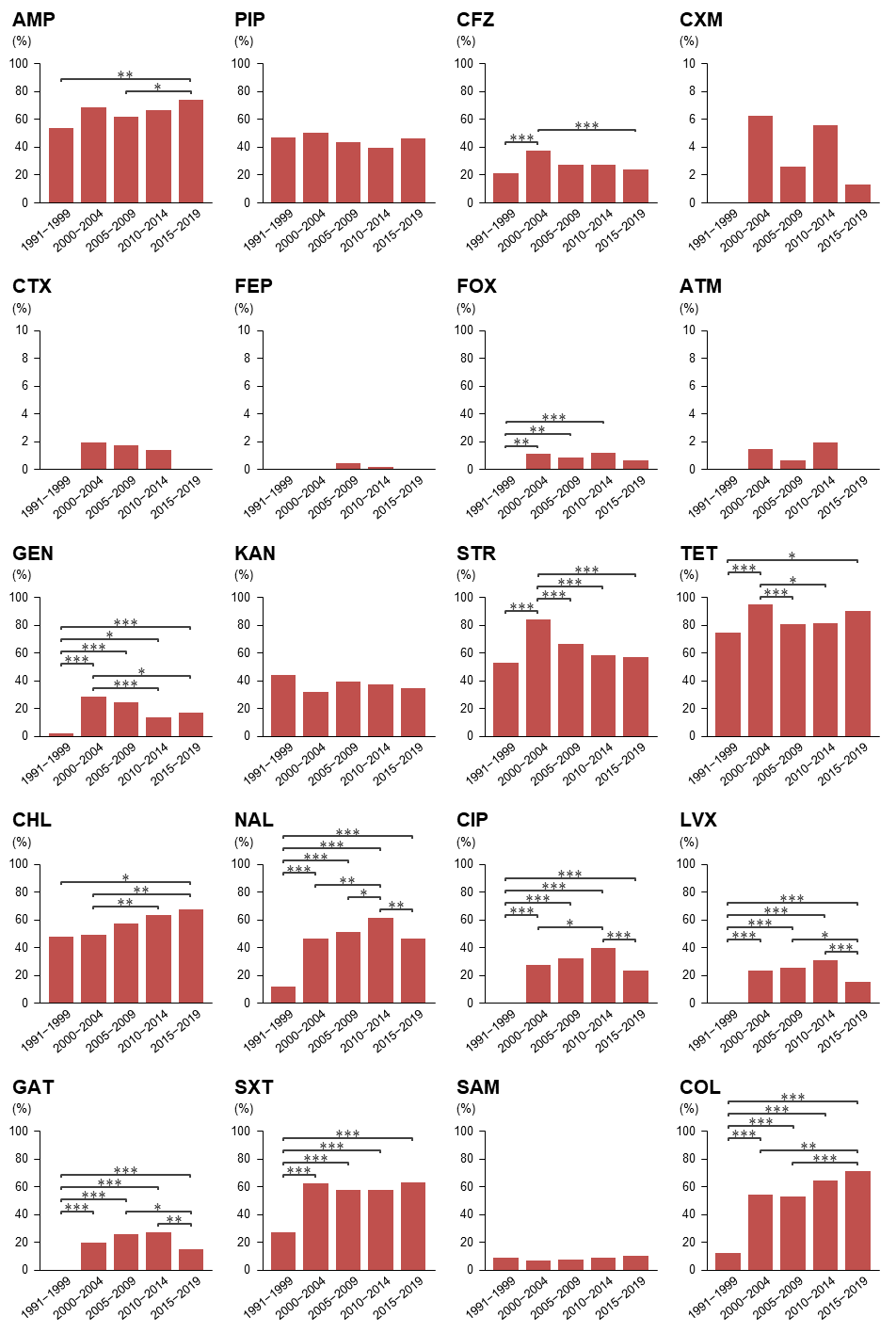


**Supplementary Figure 3.** Temporal changes in the rates of resistance to different antimicrobials. Asterisks indicate statistically significant differences (Fisher's exact test with Bonferroni correction; *, P < 0.05; **, P < 0.01; ***, P < 0.001). Abbreviations for the antimicrobials are listed in the legend of Figure 2 in the main text.
